# Intracranial Dural Arteriovenous Fistula Presenting With Bulbar and Myelopathic Features: Case Report and Literature Review

**DOI:** 10.7759/cureus.10934

**Published:** 2020-10-13

**Authors:** Bethan L Clayton, Adrian Rata, Andrew F Alalade, Nihal Gurusinghe

**Affiliations:** 1 Department of Neurosurgery, University of Manchester Medical School, Manchester, GBR; 2 Department of Neurosurgery, Royal Preston Hospital, Preston, GBR

**Keywords:** dural arteriovenous fistula, intracranial, myelopathy, angiogram, surgical disconnection, cognard

## Abstract

Intracranial dural arteriovenous fistulas are defined as pathological anastomoses between meningeal arteries and dural venous sinuses or cortical veins. Rarely, they could drain into the venous system in and around the craniocervical junction and cause myelopathy and/or bulbar features.

Due to the clinical and radiological features, prompt diagnosis poses a challenge, as there are several neurological differential diagnoses. Several mechanisms for the presentation have been considered, such as venous hypertension, direct compression by enlarged veins, and ischaemia due to infarcts or due to arterial steal. Digital subtraction angiography (DSA) is the gold-standard diagnosis for dural arteriovenous fistulas, where the visualisation of feeding arteries and the characterisation of venous drainage allows diagnosis and grading. Treatment options include conservative management, invasive (microsurgery) or minimally invasive options (transarterial or transvenous embolisation). We present a 32-year-old male who presented with myelopathy and bulbar features. Angiography demonstrated an intracranial dural arteriovenous fistula, which was successfully treated surgically.

## Introduction

Intracranial dural arteriovenous fistulas (DAVFs) can be defined as pathological anastomoses connecting meningeal arteries and dural venous sinuses and/or cortical veins [1]. They can occur either spinally or intracranially [2]. In terms of pathophysiology, DAVFs are often thought to arise de novo [1]. A minority, however, do arise from infection (mastoiditis), previous craniotomy, tumours, traumatic brain injury or dural venous sinus thrombosis [1]. Rarely, intracranial DAVFs can present with perimedullary venous drainage, resulting in an aggressive neurological course where the patient demonstrates bulbar and myelopathic features [2-3].

In these circumstances, the clinical presentation may be of progressive myelopathy with sudden deterioration/fluctuation of symptoms [4]. A myelopathic presentation of DAVFs can lead to misdiagnosis and delays in definitive treatment, with cervical transverse myelitis, encephalitis, neoplastic conditions, neuromyelitis optica and Guillain-Barré syndrome all being potential differential diagnoses [4]. The pathophysiology of myelopathy is uncertain, although multiple theories explored later in this paper may explain the phenomenon. We describe a case of intracranial DAVF causing significant acute, fluctuant myelopathy and bulbar signs.

## Case presentation

A 32-year-old man with no significant past medical history presented to the hospital with a four-week history of lower limb unsteadiness. He also complained of urinary retention, and the bladder scan showed a volume greater than one litre. Lumbar spine magnetic resonance imaging (MRI) was normal, and he was treated with intermittent self-catheterisation. However, over the course of a week, his symptoms significantly deteriorated - bilateral leg weakness and a further deterioration in balance. Upon readmission, horizontal diplopia was present alongside bilateral 2/5 lower limb weakness. He further developed upper limb and respiratory muscle weakness requiring intubation and mechanical ventilation. A whole-spine MRI was performed, which showed no abnormal signal change or contrast enhancement. After treatment with methylprednisolone for five days, he was extubated before completing seven days of plasma exchange. 

**Figure 1:**
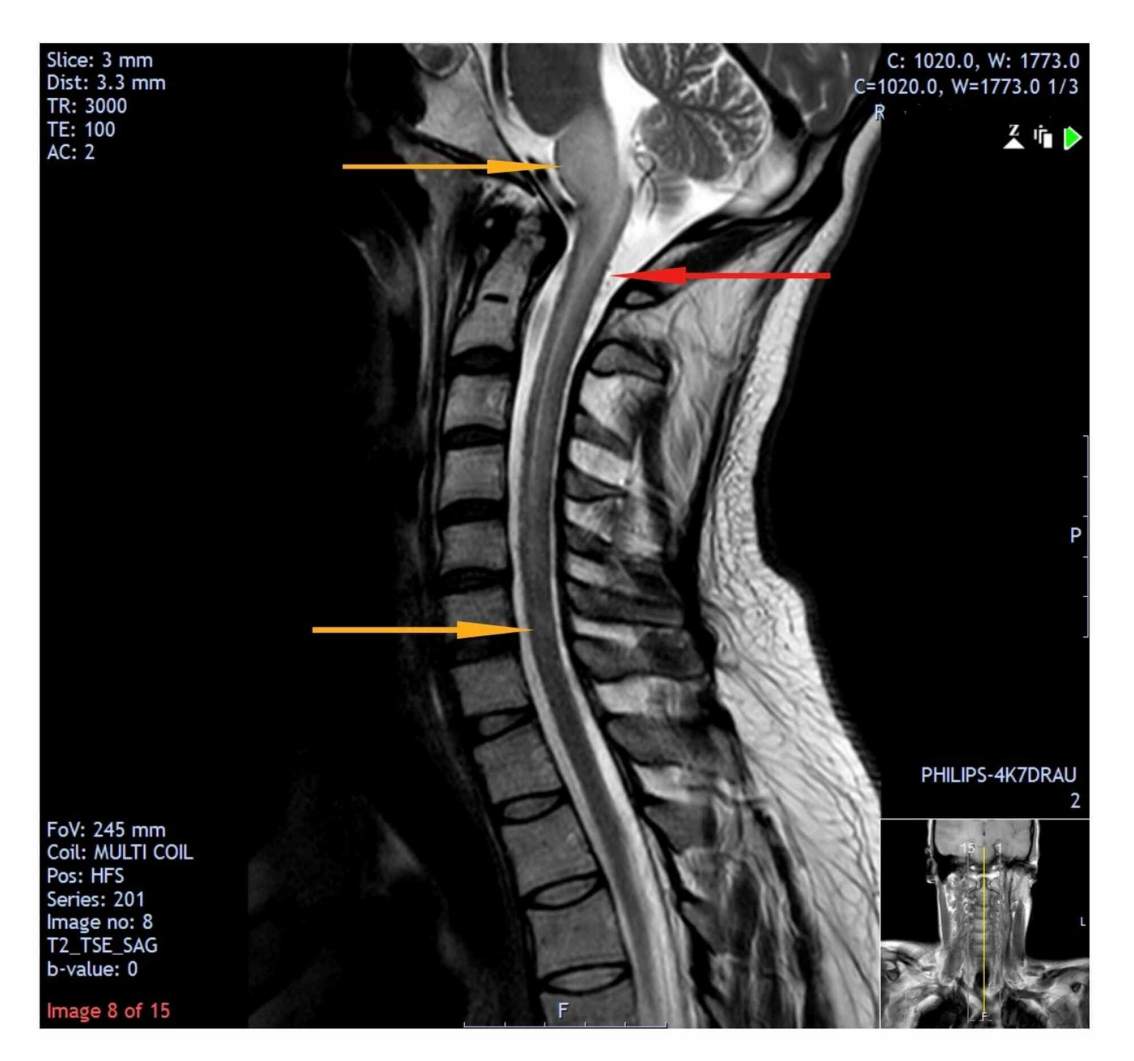
T2-weighted MRI scan showing the extent of spinal cord oedema (orange arrows) Signal change seen especially in the medulla and upper cervical cord (red arrow). This could be an indirect finding to suggest the presence of a dural arteriovenous fistula.

Several neurological conditions were considered as part of the differential diagnosis, including Guillain-Barre syndrome. Despite plasma exchange, his neurological condition deteriorated and he progressed to develop dysphonia, dysarthria and dysphagia, at which point he was transferred to our institution. Neurological examination showed he had diplopia on horizontal gaze, impaired convergence, left lateral gaze nystagmus, reduced left facial sensation, left palatal paralysis, absent jaw jerk, increased tone of the lower limbs with bilateral clonus, reduced upper limb power of 4/5 and lower limb 0-2/5, with a sensory level to pin-prick up to T5. Further investigations were performed, including serum and cerebrospinal fluid (CSF) analysis, which was negative for antibodies and oligoclonal bands and hence normal. Computed tomography (CT) of his thorax, abdomen and pelvis was unremarkable. Some improvement in lower limb power was seen with cyclophosphamide and a reducing-regimen of oral prednisolone. A repeat MRI, however, showed worsening signal changes within the brainstem and cervical spine. Prominent flow voids and spinal veins in the anterior and posterior surface of the cord with post-contrast enhancement were demonstrated (Figure 1).

**Figure 2:**
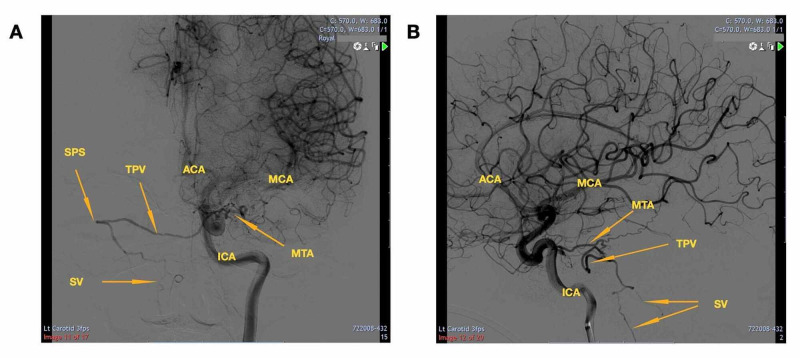
A) Anteroposterior and B) lateral views of the left internal carotid artery (ICA) Orange arrows display the course of the DAVF with relevant vessels labelled in yellow. The left internal carotid artery angiogram showed dural AVF on the left petrosal sinus with the draining vein traversing across the midline onto the opposite petrosal sinus and further down into the spinal veins. The dural AVM was supplied by the tentorial branch of the internal carotid artery. ICA (internal carotid artery), SPS (superior petrosal sinus), TPV (transverse pontine vein), ACA (anterior cerebral artery), MCA (middle cerebral artery), MTA (middle temporal artery), SV (spinal veins), AVM (arteriovenous malformation)

The resulting digital subtraction angiography (DSA), which can be seen in Figure 2, revealed a DAVF on the left petrosal sinus, which appeared to fill during the late venous phase. The DAVF originated from the left cavernous ICA towards the junction of the tentorium cerebelli and petrous ridge before draining into the spinal venous system. A final diagnosis of a left petrous apex dural arteriovenous fistula was made.

**Figure 3:**
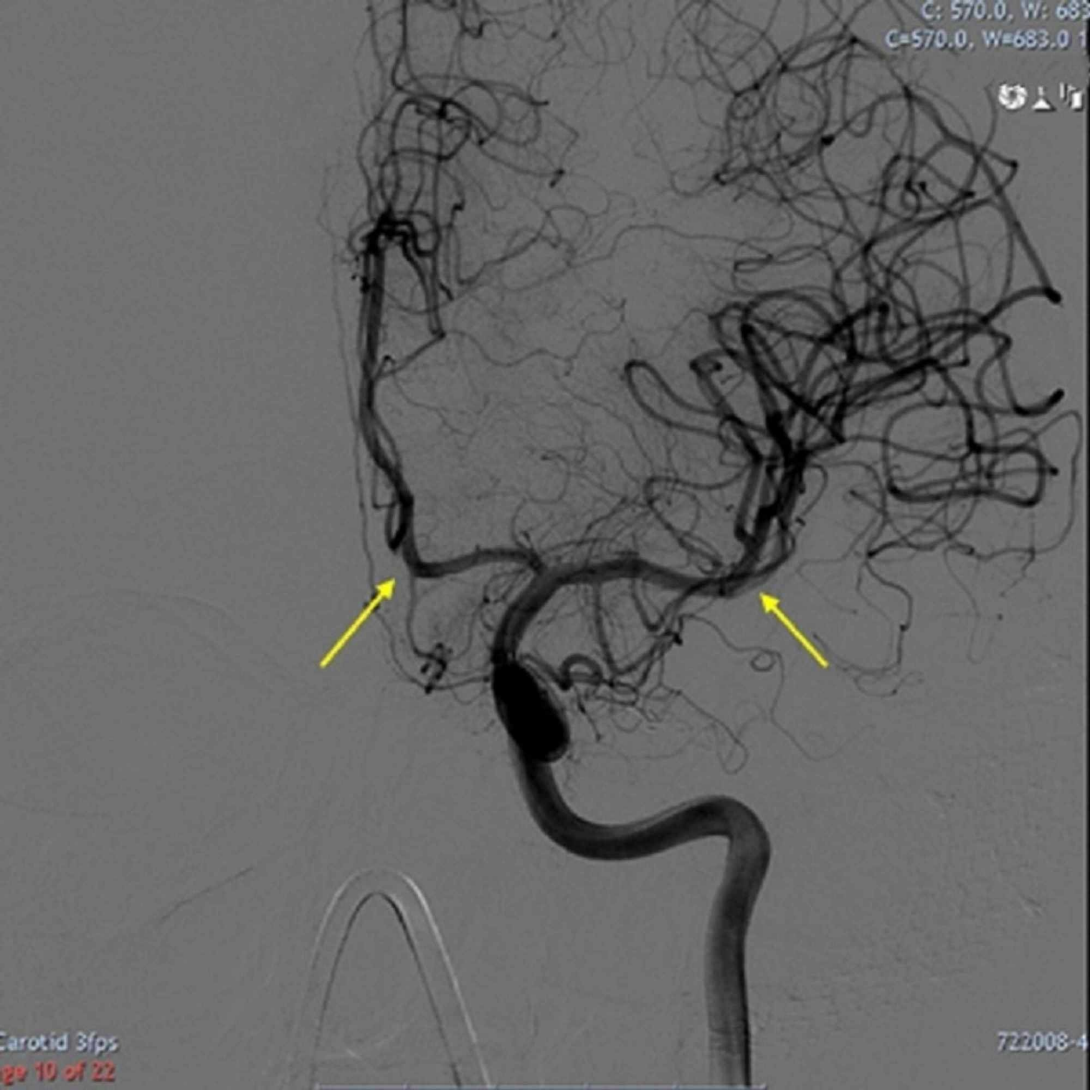
The spinal veins are not seen due to motion artefact; however, the anterior spinal veins (yellow arrows) appear to fill on late venous phase The left petrous dural AVM with spinal venous drainage is completely obliterated. AVM (arteriovenous malformation)

Embolisation was performed with the aim of occluding the arteriovenous malformation (AVM). However, a repeat DSA confirmed the filling of the draining vein. Surgical clipping with three MicroClips was applied to the draining vein and was performed with post-op DSA showing no evidence of residual AVF. Twelve months postsurgery, he had a noticeable improvement in the upper limbs and respiratory function after neurorehabilitation, and no recurrence on subsequent DSAs (Figure 3).

**Figure 4:**
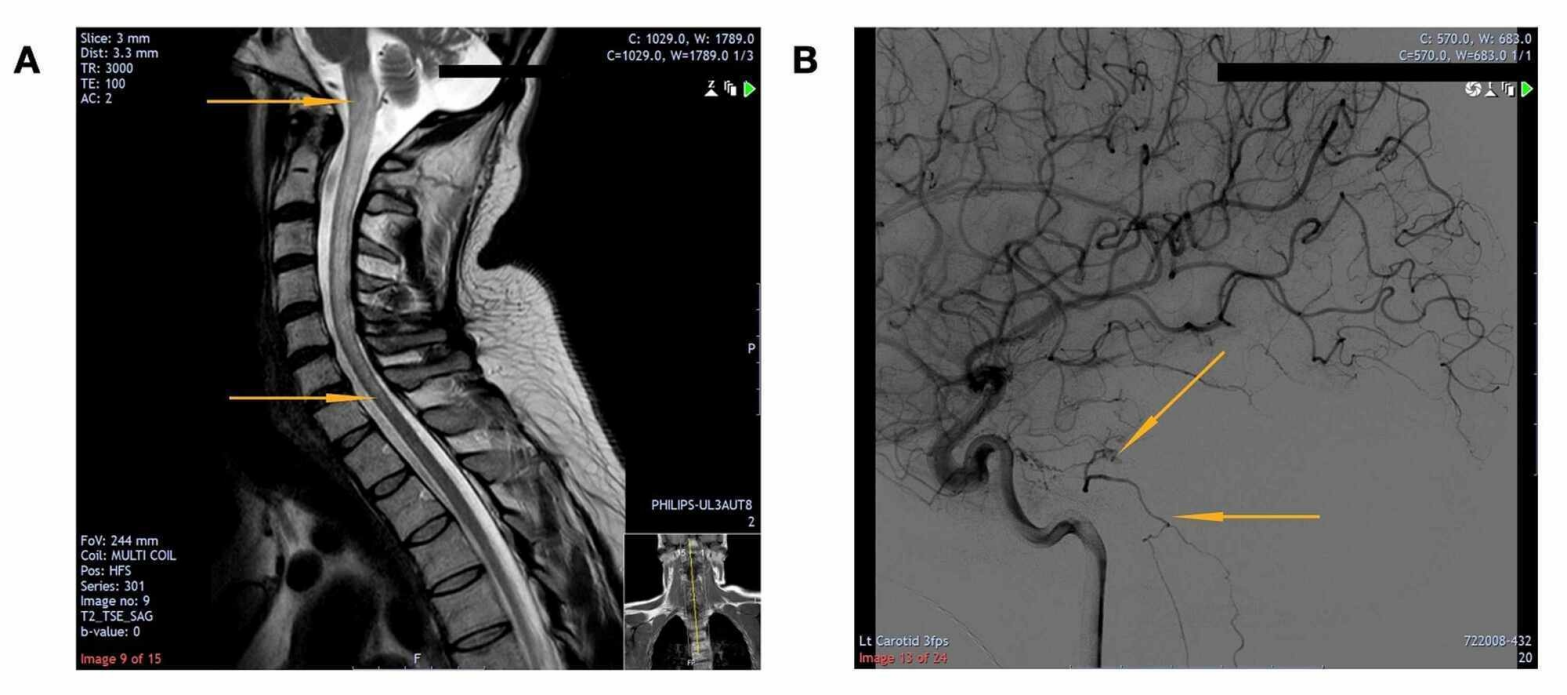
(A) Sagittal T2-weighted MRI scan showing worsening signal changes (orange arrows) and (B) DSA showing recurrence of the previously treated dural AVF (orange arrows) MRI (magnetic resonance imaging), DSA (digital subtraction angiography), AVF (arteriovenous fistula)

Unfortunately, despite remaining stable for four years post-surgery, he re-presented with worsening bilateral upper limb weakness and impaired respiratory effort. A repeat MRI showed persistent brainstem and cervical cord oedema, and a repeat DSA confirmed a recurrence of the AVM in the left petrosal sinus (Figure 4). This time, however, the arterial supply that fed the AVM arose from the meningohypophyseal/dural branch from the left cavernous internal carotid artery before draining via the superior petrosal vein and then the transverse pontine vein into the anterior and posterior spinal veins.

**Figure 5:**
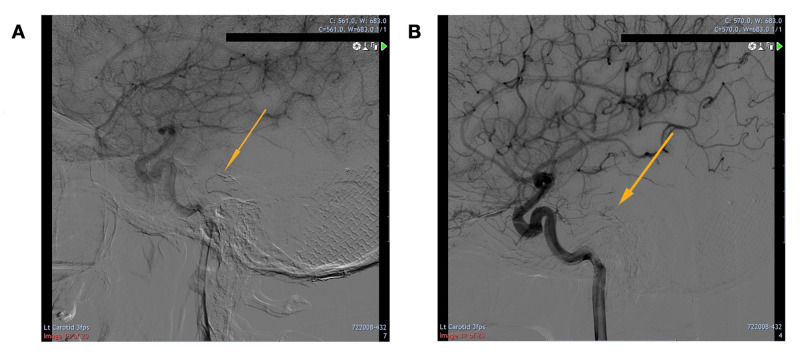
(A) Inter-op DSA showing marker clips on the draining pre-pontine vein (orange arrow) and (B) Post-op DSA showing occlusion of the draining vein and resolution of the DAVF (orange arrow). DSA (digital subtraction angiography), DAVF (dural arteriovenous fistula)

Re-exploration of the AVF followed in two stages. Initially, the suspected draining vein was clipped and confirmed on DSA. This was then occluded with two aneurysm clips in the second stage (Figure 5). Treatment alongside intensive neurorehabilitation was successful, and our patient was discharged neurologically stable, as he was back to his baseline level of fitness. 

## Discussion

Clinical presentation and classification

The classification of DAFVs can be via the Cognard grading system, which is based on the following parameters: the direction of dural sinus drainage, presence of cortical venous drainage, and method of venous outflow - spinal perimedullary veins, ectatic cortical veins or non-ectatic cortical veins [3]. The higher the grade, the increasing the severity of fistula and aggression of symptoms, as can be seen in Table 1 [5]. We described a case of Cognard Type V in this report. There is also the Borden classification, but this does not assess the direction of flow or presence of venous ectasia [6].

**Table 1:** Cognard classification system for dural arteriovenous fistulas Source [5]

Type	Definition	
I	Located in the main sinus, normal anterograde flow into dural venous sinus	
II	Located in the main sinus	IIa) With retrograde flow into the sinus
		IIb) with retrograde flow into cortical veins
		IIa+b) Retrograde flow into the sinus AND the cortical veins
III	With direct cortical venous drainage without venous ectasia	
IV	With direct cortical venous drainage with venous ectasia	
V	With spinal venous drainage	

Complications of dural AFVs include intracranial hypertension, haemorrhage and progressive myelopathy [5]. The incidence of related complications and the degree of severity increases with the Cognard type [5]. Type I DAVFs follow a typically benign course due to normal anterograde flow in the dural venous sinus [7]. Types IIa and b can be more aggressive due to retrograde flow into the cortical veins and venous sinus [7]. IIa has a 20% incidence of intracranial hypertension, whereas IIb has a 10% risk of haemorrhage due to venous reflux [7]. IIa+b have a more aggressive nature, and the risk of haemorrhage is reported to be about 66% [7]. Type III and IV have a 40% and 65% risk of haemorrhage, respectively [7]. Finally, due to their direct drainage into spinal perimedullary veins, it is type V DAVFs that can present with acute myelopathy and/or bulbar features and lead to neurological mimics leading to the aforementioned diagnostic uncertainty, and as seen in our patient discussed in this report [7].

Diagnostic imaging and treatment

Regarding diagnostic evaluation, both CT and MRI scans can initially detect DAVFs by demonstrating dilated vessels and sinus enlargement or occlusion [7]. Characteristic findings on MRI are of diffuse hyperintensity from vasogenic oedema surrounding the cord [8]. On T2-weighted imaging, there may be vascular flow voids and patchy gadolinium enhancement [8]. However, the DSA is considered the gold standard in both diagnosis and treatment planning, due to its superior sensitivity in characterising DAVFs. Angiography allows the fistula to be defined, determines sinus patency and delineates affected dilated veins [7]. This not only allows the complete mapping and assessment of the treatment approach, but it also allows the confirmation of AVF obliteration post-surgery [9].

Multiple treatment modalities are available for DAVFs, all of which aim for complete disconnection of the venous drainage [7]. Treatment decisions are based on grade, the severity of symptoms and the risk versus benefits of the administered intervention [10]. Low Cognard grade DAVFs that follow a benign course could be managed conservatively [11]. Where treatment is indicated, stereotactic radiosurgery is a management option for either low-grade symptomatic lesions, DAVFs with unfavourable anatomy and angioarchitecture, or patients with significant medical co-morbidities who might not be able to tolerate more invasive procedures [11]. However, for the higher grade and symptomatic DAVFs as seen in the patient in this study, microsurgical disconnection or endovascular embolization are viable management options [11]. Multi-modality treatment options are also available that combine the aforementioned treatment methods, as seen in our patient.

Both minimally invasive and non-minimally invasive treatment options exist, however, a consensus regarding the best modality does not. The relative rarity of DAVFs means large studies in the literature regarding their management are yet to be performed [12]. Minimally invasive options to treat DAVFs include transarterial embolisation (TAE) or transvenous embolisation (TVE) or a combination of both [13]. The goal of TAE is to use Onyx® to penetrate the fistula and completely embolise the fistula [13]. If this cannot be achieved due to the DAVF possessing multiple fistula points, balloon-assisted TAE alongside Onyx® embolisation may be used [13]. TVE can be performed either via coil packing of the fistula, Onyx® embolisation or via balloon-assisted Onyx® TVE [13]. Micro-surgery was used in this case, after the check DSA confirmed non-resolution of the DAVF. Surgical disconnection is not minimally invasive but is usually necessary in select cases [12]. The role of microsurgery has been suggested by Wachter et al. to be reserved for either the early management of aggressive DAVFs or as an adjunct alongside endovascular therapy in the cases of incomplete obliteration after Onyx® embolisation [12,14].

Diagnostic uncertainty: neurological mimics

As mentioned in our case and various other reports in the literature, type V DAVFs tend to present insidiously, mimicking other neurological conditions such as cervical transverse myelitis, Guillain-Barre syndrome, encephalitis, neoplastic conditions or neuromyelitis optica [2]. Different pathophysiological factors responsible for DAVFs presenting as acute myelopathy and/or bulbar palsy have been hypothesised in the literature. The proposed theories range from venous hypertension, direct compression by enlarged veins, ischaemia due to infarcts and due to arterial steal [2]. However, the disappearance of clinical and imaging changes after disconnection of the venous drainage in most cases of DAVFs makes venous hypertension the more likely theory for the neurological deficits [15]. This phenomenon is thought to be due to the AVF affecting the normal venous drainage of spinal perimedullary veins [10]. The effect of this impairment of normal drainage causes high-pressure venous drainage at the cord surface, causing venous congestion, hence venous hypertension [10]. Consequently, this results in vasogenic oedema and resulting focal neurological deficits [10]. This mechanism is also suggested to be the reason behind the rarely reported worsening of the clinical condition in patients treated with corticosteroids for their myelopathy [10]. This is due to transient fluid retention caused by corticosteroids, causing venous engorgement and exacerbating spinal cord oedema [16]. This leads to neurological worsening, which may improve upon the cessation of corticosteroids or lead to a permanent deficit. Although our patient did not experience acute worsening in his clinical condition with corticosteroid administration, there was a progressive signal change on MRI post-steroids. This phenomenon has also been reported in up to 50% of patients with spinal DAVFs treated in this manner, with the clinical deterioration not always transient and recoverable upon the cessation of corticosteroids [3]. This further highlights the importance of determining the definitive cause of myelopathy prior to initiating empirical steroid treatment.

## Conclusions

Intracranial DAVFs are rare, and their presentation can be complex. This case highlights the challenges of diagnosing intracranial DAVFs, and myelopathic symptoms that fail to respond to standard treatment should lead to a high index of suspicion. The clinical presentation of DAVFs ranges from symptoms of haemorrhage to progressive myelopathy with a broad, non-specific presentation. There should be a low threshold for a repeat MRI or progressing to DSA when faced with a patient who fails to improve, or further deteriorates, despite commencing treatment for myelopathy. The successful treatment of DAVFs should aim for the complete disconnection of venous drainage. Treatment decisions are based on symptom severity and the grade of DAVF, with improving functional outcomes possible after definitive management.
